# Hearing Outcomes and Complications of Cochlear Implantation in Elderly Patients over 75 Years of Age

**DOI:** 10.3390/jcm10143123

**Published:** 2021-07-15

**Authors:** Rie Kanai, Shin-ichi Kanemaru, Kaoru Tamura, Yoshiko Noda, Naomi Umezawa, Misaki Yoshida, Toru Miwa, Tomoya Yamaguchi, Shinichiro Kita, Akiko Kumazawa, Hiroyuki Harada, Toshiki Maetani

**Affiliations:** Department of Otolaryngology, Head & Neck Surgery, Tazuke Kofukai Medical Research Institute, Kitano Hospital, Osaka 530-8480, Japan; riekanai.ent@gmail.com (R.K.); ka-tamuta@kitano-hp.or.jp (K.T.); y-noda@kitano-hp.or.jp (Y.N.); n-shibata@kitano-hp.or.jp (N.U.); misaki-yamamoto@kitano-hp.or.jp (M.Y.); miw.1101com@gmail.com (T.M.); t_yamaguchi@ent.kuhp.kyoto-u.ac.jp (T.Y.); shinichirou-kita@kitano-hp.or.jp (S.K.); a-kumazawa@kitano-hp.or.jp (A.K.); h_harada@ent.kuhp.kyoto-u.ac.jp (H.H.); t-maetani@kitano-hp.or.jp (T.M.)

**Keywords:** cochlear implant, elderly, 75 years, hearing outcomes, complications

## Abstract

Objective: Populations are aging in many countries, and the proportion of elderly people with severe to profound hearing loss is increasing in parallel with the increasing average life span. The objective of this study was to investigate the outcomes of cochlear implant (CI) surgery in elderly patients compared to those in younger patients. Methods: The outcomes of CI surgery were retrospectively investigated for 81 adults (32 men and 49 women) who underwent CI surgery at our hospital. They were divided according to age at the time of implantation into the younger group (<75 years of age; *n* = 49) or elderly group (≥75 years of age; *n* = 32). Results: The mean sentence recognition score on the CI-2004 Japanese open-set test battery (±standard deviation) was 82.9% ± 24.1 in the younger group and 81.9% ± 23.2 in the elderly group, with no significant difference between the groups (Mann–Whitney U test). The incidence of major complications that required surgical treatment was not significantly different between the groups (4.1% vs. 6.2%, respectively). Thus, there were no severe complications that could affect general health status in either group. Three patients in each group died for reasons unrelated to CI surgery during follow-up. The proportion of patients who were alive and continued to use the CI five years after surgery was 92.8% and 91.5%, respectively. Conclusion: Our results show good speech recognition and a low incidence of major complications in elderly patients. This comprehensive report on the outcomes of CI surgery in elderly patients will be helpful to the elderly with severe to profound hearing loss when deciding whether to undergo CI surgery.

## 1. Introduction

Populations are now aging in many countries around the world, particularly in Japan. The average life expectancy in Japan is 81 years for men and 87 years for women [1]. A larger elderly population results in more people with hearing loss due to aging and other factors. Moreover, hearing loss can lead to a host of problems, including an increased mortality risk, cognitive decline, and isolation and depression due to an inability to participate in conversation [2,3]. Hearing aids are often effective in elderly people with moderate hearing loss, but a cochlear implant (CI) may be required in those with severe to profound hearing loss. For example, in South Korea, where the population is aging rapidly, as in Japan, the number of patients over 80 years with severe to profound hearing loss tripled between 2006 and 2015 [4]. The same phenomenon is probably occurring in other countries with aging populations. Therefore, CI therapy is increasingly needed in elderly people with severe to profound hearing loss. However, when CI surgery is recommended, these patients and their families often express concern about surgical complications under general anesthesia, the effectiveness of the CI, and for how long they will benefit from the use of a CI because of their advanced age. Therefore, it is essential to give these patients a clear explanation of the outcomes of CI surgery.

Several reports suggest that there is no difference in hearing outcomes after CI surgery between elderly patients and their younger counterparts, and that the risk of major complications is not increased in elderly patients. The impact of cochlear implant therapy in the elderly on reducing the incidence of dementia, as well as cost effectiveness, have also been reported [5,6]. Mosnier et al. reported that if using a CI could delay the onset of dementia in the elderly with hearing loss by one year, it would reduce the incidence of dementia by 9 million cases by 2050 [5]. A study in Switzerland [6] found that CI surgery was more cost-effective than a hearing aid alone in women aged up to 91 years and men aged up to 89 years at the time of implantation.

However, in previous studies about the outcomes of CI surgery in elderly people, the cutoff age for the elderly was often set at 60–65 years [7,8,9,10,11]. Although the definition of elderly in Japan continues to be 65 years or over, recent studies show that the Japanese elderly are relatively youthful, and that those under the age of 75 years are often in good mental and physical health [12]. This trend is likely to become more widespread in the future. Therefore, the conventional definition of elderly, namely 60 or 65 years of age, may not reflect the current health status of the elderly in Japan. In recent years, the Japan Geriatrics Society has proposed that the elderly aged 65 and over are classified as pre-old age (under 74 years old) and old age (over 75 years old) [12].

In light of this situation, using a cutoff age of 75 years for the elderly, we investigated the hearing outcomes and complications after CI surgery in the elderly. In addition, we sought to determine how long elderly patients would be expected to derive benefit from using a CI. This information would be invaluable when considering CI surgery for elderly patients with severe to profound hearing loss, their families, and medical professionals involved with CI.

## 2. Materials and Methods

### 2.1. Subjects

Eighty-seven adults with bilateral severe to profound hearing loss underwent CI surgery at Kitano Hospital between May 2009 and March 2020. Two otologists performed all surgical procedures. After excluding four patients who underwent bilateral CI surgery, one who attended only one postoperative rehabilitation session after surgery, and one who had prelingual deafness, this left 81 patients for analysis in this retrospective study.

Patients were divided according to age at the time of implantation into the younger group (aged < 75 years, *n* = 49) or ≥75 years (the elderly group, *n* = 32). Preoperative chest radiographs, electrocardiograms, and the results of laboratory investigations were obtained in all patients to confirm their ability to tolerate surgery under general anesthesia.

### 2.2. Parameters Evaluated

The following information was obtained from each patient’s medical records: cause of hearing loss, the prevalence of comorbidities that could affect the perioperative status, use of antithrombotic agents, preoperative and postoperative audiometric findings, postoperative complications, and the duration of continued use of the CI. The preoperative hearing level was evaluated using the pure-tone average (500 Hz, 1000 Hz, 2000 Hz, 4000 Hz) and the maximum discrimination score on the 67-S Japanese monosyllable word list. When the pure-tone threshold was scaled out for a certain frequency, it was regarded as 115 dB HL for calculation.

Postoperative speech recognition was evaluated using the CI-2004 Japanese open-set test battery, which is performed using a female voice at a level of 60 dB in a quiet setting. The best test results obtained more than six months after surgery were analyzed. However, the results performed within six months after surgery were used for one patient who died of an unrelated cause and for another patient whose CI was removed within six months of surgery because of an infection. Complications were classified as major if they required surgery or caused a deterioration in overall health status and minor if they produced only transient symptoms or improved with local treatment only. Dizziness and vertigo that improved within a week were not included as complications. Based on the follow-up period for each patient, the percentage of patients who were alive and continued to use their CI was examined over time.

### 2.3. Devices

Patients used the following types of devices: Med El PULSAR, Med El SONATA, Med El CONCERT, Med El SYNCHRONY, Cochlear Contour Advanced, Cochlear CI 422, Advanced Bionics HiRes 90 K, and Advanced Bionics HiRes 90 K Advantage.

### 2.4. Statistical Analysis

The data for preoperative pure tone average, maximum discrimination score, postoperative sentence recognition score, and monosyllable recognition scores are shown as the mean ± standard deviation. Since these data were not normally distributed in the Shapilo–Wilk test, the Mann–Whitney U test was used to compare these date between the two groups. The ratio of gender, ratio of laterality, prevalence of comorbidities, use of antithrombotic agents, and the incidence of postoperative complications were compared between the two groups using the chi-squared test and Fisher’s exact test. A *p*-value < 0.05 was considered statistically significant. The correlation between age at implantation and sentence recognition score was examined using the Spearman rank correlation coefficient test. Kaplan–Meier curves were used to show the changes in the proportion of patients who were alive and continued to use their CI over time. The curves were compared between the two groups using the log-rank test. All statistical analyses were performed using Excel Tokei 2012.

## 3. Results

### 3.1. The Demographic and Clinical Characteristics

The demographic and clinical characteristics of each group are shown in Table 1. There was a significant difference in the overall prevalence of comorbidities that could have affected perioperative status (38.8% in the younger group and 62.5% in the elderly group). The prevalence of hypertension, diabetes mellitus, and heart disease tended to be higher in the elderly group than in the younger group. The percentage of patients taking antithrombotic agents was significantly higher in the elderly group than in the younger group (24.1% vs. 6%). Antithrombotic drugs were discontinued or changed in the perioperative period to avoid excessive bleeding during surgery. There was no significant difference in the preoperative pure-tone average and maximum discrimination score between the two groups.

### 3.2. Hearing Outcome

**Figure 1:**
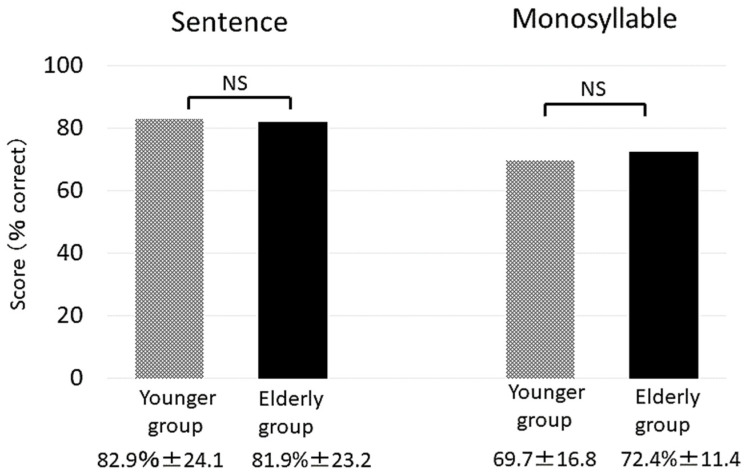
Postoperative sentence and monosyllable speech recognition results (% correct) on the CI-2004 in a quiet setting.

There was no significant difference in the preoperative pure-tone average and maximum discrimination score between the two groups. Figure 1 shows the postoperative speech recognition results obtained in a quiet environment. In the postoperative period, the mean sentence recognition score was 82.9% ± 24.1 in the younger group and 81.9% ± 23.1 in the elderly group; the respective mean monosyllable recognition scores were 69.7% ± 16.8 and 73.9% ± 20.2, respectively. There was no significant difference in sentence and monosyllable recognition scores between the two groups. The sentence recognition score in each patient was not correlated with age at implantation.

### 3.3. Postoperative Complications

The incidence of postoperative complications is shown in Table 2. One patient in each group required revision surgery due to cord exposure. Cholesteatoma occurred in one elderly patient, and a severe middle infection occurred in one younger patient. The CI was removed in both patients. The incidence of major complications requiring reoperation or removal of the CI was not significantly different between the younger and elderly groups (4.1% vs. 6.4%). Thus, no serious complications could lead to deterioration in general health status in either group. However, the incidence of minor complications was significantly higher in the elderly group (12.8% vs. 31.1%). Skin problems, including pain and redness at the receiver/stimulator (RS) site, were observed in several elderly patients. In addition, the incidence of dizziness and vertigo tended to be higher in the elderly group.

### 3.4. Changes in the Percentage of CI Users

**Figure 2:**
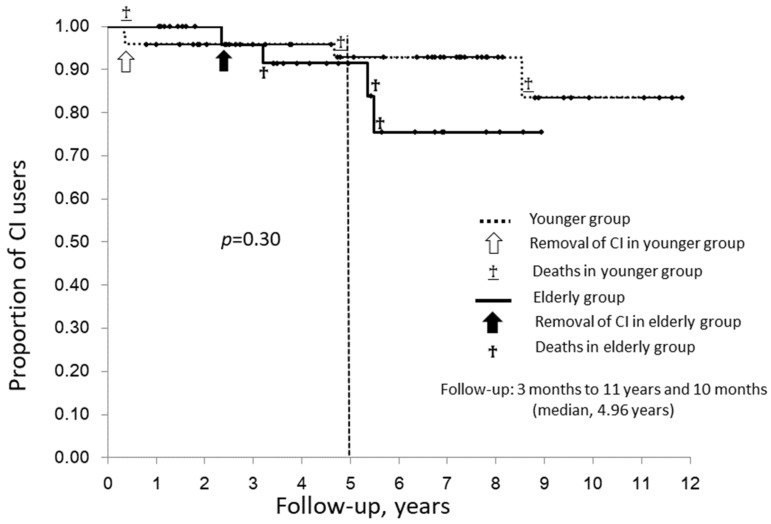
Kaplan–Meier curve showing time from CI surgery to cessation of cochlear implant use in younger patients versus elderly patients.

Changes in the percentage of patients who were alive and continued to use their CI over time (referred to as CI users here) are shown as a Kaplan–Meier curve in Figure 2. A log-rank test showed no significant difference in the percentage of CI users over time between the two groups. Based on the median postoperative observation period for all 81 patients of about five years, CI users at five years after surgery amounted to 92.8% of the younger group and 91.5% of the elderly group.

## 4. Discussion

We examined the perioperative and postoperative course of CI surgery in elderly people (aged ≥ 75 years) and compared the outcomes with those in younger patients (aged < 75 years).

### 4.1. Hearing Outcome

In many reports on CI use in the elderly population, the cutoff age for separating the elderly from the non-elderly was set at 60–70 years of age [7,9,10,11,13,14,15,16]. In this study, even though the cutoff age of the elderly was older than in previous reports, speech recognition results in elderly patients under quiet conditions were as good as that in non-elderly patients (Figure 1). Many studies have reported dramatic improvement in speech recognition after CI surgery in elderly patients [7,8,9,10,13,14,17,18]. They have shown that hearing under quiet conditions was not significantly different from non-elderly patients [7,11,14,16]. Our results are consistent with these reports.

Although we only had the date of hearing ability in a quiet condition due to a lack of equipment in our facility for sound field tests in noisy condition, it has been widely reported that speech recognition in a noisy environment is more deficient in the elderly than in the non-elderly [9,14,15,18]. This finding is attributed to the decline in the central nervous system and cognitive function related to auditory processing under noisy conditions in the elderly. Therefore, they may have difficulty recognizing conversations with multiple people or in noisy environments, even with a CI. However, our finding of good speech recognition in elderly patients with a CI in a quiet environment suggests that a CI can contribute greatly to improving communication during one-on-one conversations, especially with close family members and caregivers, and reduce the burden on both parties [8].

### 4.2. Postoperative Complications

The prevalence of comorbidities and patients using antithrombotic medication were significantly higher in the elderly group than in the younger group. However, there were no postoperative complications that could deteriorate overall health status, and the incidence of major complications was very low in the elderly patients. Coelho et al. [19] reported that perioperative complications were significantly higher in patients over 70 years of age who underwent CI surgery if their American Society of Anesthesiologists physical status was 3–4 than if it was 1–2. However, the percentage did not increase with age. In our study, any comorbidities in elderly patients were appropriately managed with medication. Therefore, age and comorbidities are not risk factors for complications per se; the severity of comorbidities affects complications. In other words, CI surgery can likely be performed safely even in elderly patients if their preoperative evaluation indicates that they are in good general condition.

The incidence of minor complications was significantly higher in the elderly group. The incidence of dizziness, vertigo, skin redness, and pain around the RS tended to be higher in the elderly group. Several reports indicate that equilibrium problems after CI surgery are significantly more common in elderly patients [17,20,21]. It has been suggested that compensatory vestibular function, cognitive function, peripheral proprioception, and muscle weakness may contribute to poor balance postoperatively in this age group [21]. Therefore, rehabilitation for equilibrium function should be started as soon as possible after surgery in elderly patients who complain of dizziness or vertigo. In addition, skin inflammation around the RS is more likely to occur in elderly patients because of age-related thinning of the skin on the scalp [22]. Inflammation of the scalp may cause necrosis at this site, which prevents patients from using their CI. However, it is difficult for elderly patients to notice this themselves. Therefore, family members and medical staff should be instructed to monitor the patient’s surgical site carefully [22,23]. If redness or pain occurs on the skin around the RS site, the magnet’s strength should be changed as soon as possible to prevent skin damage.

### 4.3. The Possibility of Less Invasive CI Surgery for Elderly Patients

CI surgery using an approach without mastoidectomy has been reported [24,25]. We also have experience taking this approach in some cases. This approach has the benefit of shortened time of surgery [24]. It may be an option as a less invasive surgical method for the elderly. In this method, however, there are some risks of damaging the chorda tympani nerve and exposing the electrode cord during the several years after the operation.

### 4.4. Prognosis in Elderly Patients after CI Surgery

At our hospital, patients continue to attend outpatient visits for hearing rehabilitation after CI surgery as long as they do not self-interrupt regular appointments. We check whether they can use their CI daily. In this study, there was no significant difference between the two groups in the percentage of patients who continued to use their CI without complications that required CI removal, without death occurring, during the follow-up period (Figure 2). At five years postoperatively, the median observation period, the percentage of elderly patients who continued to use a CI was 91.5%, similar to that in younger patients (Figure 2). Currently, the average life expectancy in the Japanese population is 81 years for men and 87 years for women. In this study, the mean age at implantation in elderly patients was 80.8 years, almost the same as the average life expectancy in men and close to the average life expectancy in women. Our results suggest that these elderly patients can continue to use a CI for more than five years after implant surgery. Considering their age and average life expectancy, this period would not necessarily be short.

### 4.5. The Expected Effect of CI in the Elderly

Hearing loss has been identified as one of the factors that accelerate the decline of cognitive function [26]. This may be because most of the mental resources are spent on auditory perceptual processing at the expense of other cognitive functions, such as working memory, and opportunities for contact with others are reduced due to social isolation and depression caused by hearing loss. Cognitive function usually declines with age; however, a previous study reported that it was stable before and after CI surgery in elderly patients, with no cases of deterioration [27] and improvement in some cases [5]. This is thought to be because the ability to communicate with others when using a CI reduces the cognitive load during listening and helps to alleviate depression, which is a factor in cognitive decline [5]. Our study has shown that even people 75 years or over have good speech recognition after CI surgery, and are likely to continue using their CI for an extended period. Therefore, we guess that CI therapy for elderly patients with severe to profound hearing loss could contribute to reduce or delay the onset of dementia in them.

## 5. Limitations

This study has several limitations. First, our study does not have data for an age-matched control group. Therefore, we could not compare speech recognition outcomes between patients with age-appropriate hearing and those with cochlear implants. Second, we could not examine the differences in speech recognition between the elderly and non-elderly in a noisy environment due to equipment limitations, so we had to rely on previous reports. Third, because this was a retrospective study, the rehabilitation and examination intervals varied, and it was not possible to determine if the results for speech recognition changed over time. However, except for patients who died or were lost to follow-up, all patients continued rehabilitation with a speech therapist, so we were able to confirm whether there was a severe decline in their speech recognition. Finally, we have mentioned the effects of cochlear implant therapy on the development of dementia based on reference to the past literature. In the future, we would like to conduct a prospective study to investigate the changes in cognitive function in elderly patients with CI before and after surgery.

## 6. Conclusions

This study found that a CI can provide good speech recognition in elderly patients aged 75 years or over. Furthermore, they are likely to continue to use the CI for a long time, considering their average life expectancy. Although the proportion of patients with comorbidities was higher in our elderly group than in our younger group, no complications led to the deterioration of general health status. The incidence of serious complications requiring removal of the CI was very low in elderly patients. Our findings indicate that CI surgery is a safe procedure for elderly patients when comorbidities are managed appropriately. Age alone should not be a contraindication to CI surgery.

This comprehensive report on the perioperative and postoperative outcomes of CI surgery in elderly subjects will be helpful to the elderly with severe to profound hearing loss and their families, as well as the medical professionals caring for them when deciding whether to undergo CI surgery.

## Figures and Tables

**Table 1:** Patient demographic and clinical characteristics.

	Younger Group (*n* = 49)	Elderly Group (*n* = 32)	*p*-Value
Gender Male:Female	22:27	10:22	0.21
Age at implantation (years), mean ± SD	56.7 ± 15.4	80.8 ± 3.2	
Laterality of implanted ear, Right:Left	23:26	17:15	0.58
Follow-up duration (years), median ± SD	6.6 ± 3.2	4.0 ± 2.4	
Preoperative PTA (dB HL) mean ± SD			
Implanted ear	103.1 ± 12.8	100.5 ± 14.4	0.54
Contralateral ear	99.2 ± 16.2	92.7 ± 17.9	0.08
Maximum discrimination score (%) mean ± SD			
Implanted ear	13.9 ± 16.0	12.1 ± 17.6	0.16
Contralateral ear	26.3 ± 24.3	20.4 ± 23.3	0.12
Type of implant (*n*)			
Med-El	28 (57.1%)	22 (68.8%)	
Cochlear	17 (34.7%)	9 (28.1%)	
Advanced Bionics	4 (8.1%)	1 (3.1%)	
Etiology (*n*)			
Unknown	22 (44.9%)	8 (25%)	
Chronic otitis media	8 (16.3%)	13 (40.6%)	
Congenital hearing impairment	4 (8.1%)	0	
Otosclerosis	3 (6.4%)	0	
Idiopathic sensorineural hearing loss	2 (4.0%)	6 (18.8%)	
in only hearing ear			
Meningitis	2 (4.0%)	1 (3.1%)	
Viral labyrinthitis	2 (4.0%)	0	
Eosinophilic otitis media	2 (4.0%)	0	
Auditory neuropathy	1 (2.0%)	3 (9.4%)	
Meniere’s disease	1 (2.0%)	1 (3.1%)	
Other	2 (4.0%)	0	
Prevalence of comorbidity (*n*)	19 (38.8%)	20 (62.5%)	0.0000007
Hypertension	6 (12.2%)	6 (18.8%)	
Diabetes	3 (6.1%)	6 (18.8%)	
Cardiac disease	1 (2.0%)	6 (18.8%)	
Cerebrovascular disease	4 (8.2%)	4 (12.5%)	
Renal failure	2 (4.1%)	2 (6.3%)	
Asthma	3 (6.1%)	2 (6.3%)	
Psychiatric illness	2 (4.1%)	1 (3.1%)	
Epilepsy	2 (4.1%)	1 (3.1%)	
Other	3 (6.1%)	1 (3.1%)	
Use of an antithrombotic agent (%)	3 (6.1%)	7 (21.9%)	0.04

Younger group, aged < 75 years. Elderly group, aged ≥ 75 years. PTA, pure-tone average; SD, standard deviation.

**Table 2:** Incidence of postoperative complications.

	Younger Group (*n* = 49)	Elderly Group (*n* = 32)	*p*-Value
Major (*n*)	2 (4.1%)	2 (6.2%)	0.66
Exposure of electrode array	1 (2.0%)	1 (3.1%)	
Removal of CI for otitis media	1 (2.0%)	1 (3.1%)	
Minor (*n*)	6 (12.8%)	10 (31.3%)	0.035
Wound infection	0	1 (3.1%)	
Perforation of tympanic membrane	0	1 (3.1%)	
Retroauricular hematoma	2 (4.1%)	0	
External otitis	1 (2.0%)	0	
Skin pain and flare around the RS	0	3 (9.4%)	
Mild DVT	1 (2.0%)	0	
Persistent dizziness	0	3 (9.4%)	
Repeated vertigo attacks	1 (2.0%)	2 (6.2%)	

Younger group, aged < 75 years. Elderly group, aged ≥ 75 years. CI, cochlear implant; DVT, deep vein thrombosis; RS, receiver/stimulator.

## Data Availability

The data presented in this study are available on request from the corresponding author.
